# Transcriptomic Signatures in IgA Nephropathy: From Renal Tissue to Precision Risk Stratification

**DOI:** 10.3390/ijms262010055

**Published:** 2025-10-15

**Authors:** Charlotte Delrue, Marijn M. Speeckaert

**Affiliations:** 1 Department of Nephrology, Ghent University Hospital, 9000 Ghent, Belgium; charlotte.delrue@ugent.be; 2 Research Foundation-Flanders (FWO), 1000 Brussels, Belgium

**Keywords:** IgA nephropathy, transcriptomics, RNA sequencing, biomarkers, precision nephrology, glomerular disease, inflammation, fibrosis

## Abstract

IgA nephropathy (IgAN) is the most prevalent type of primary glomerulonephritis, with heterogeneous clinical outcomes. Conventional prognostic factors, such as proteinuria, eGFR, and Oxford histologic classification, have poor sensitivity and specificity. Recently, transcriptomic profiling has been employed to provide insights into the molecular definition of IgAN and facilitate patient stratification in those at risk of disease progression. In this review, we summarize our current understanding of IgAN derived from bulk RNA sequencing, single-cell transcriptomics, spatial transcriptomics, and gene expression profiling to elucidate the molecular characteristics of IgAN. Bulk transcriptomics of glomerular and tubulointerstitial compartments highlighted consistently upregulated genes (e.g., *CCL2*, *CXCL10*, *LCN2*, *HAVCR1*, *COL1A1*) and altered pathways (e.g., NF-κB, TGF-β, JAK/STAT, and complement) that are associated with clinical decline. Single-cell and single-nucleus RNA-sequencing has also identified the value of pathogenic cell types and regulatory networks in mesangial cells, tubular epithelium, and immune infiltrates. Furthermore, noninvasive transcriptomic signatures developed from urine and blood may represent useful real-time surrogates of tissue activity. With the advent of integrated analyses and machine learning approaches, personalized risk models that outperform traditional metrics are now available. While challenges remain, particularly related to standardization, cohort size, and clinical deployment, transcriptomics is likely to revolutionize IgAN by providing early risk predictions and precision therapeutics. Unlike prior reviews, our work provides an integrative synthesis across bulk, single-cell, spatial, and noninvasive transcriptomics, linking molecular signatures directly to clinical translation in risk stratification and precision therapeutics.

## 1. Introduction

Immunoglobulin A nephropathy (IgAN), also known as Berger’s disease, is the most common form of primary glomerulonephritis worldwide and has a highly heterogeneous clinical course. While some patients remain stable for decades, approximately 30–40% progress to kidney failure within 10–20 years of diagnosis [1]. IgAN is characterized by mesangial deposition of galactose-deficient IgA1 (Gd-IgA1)-containing immune complexes, resulting in mesangial hypercellularity, matrix expansion, inflammation, and eventual glomerulosclerosis. Conventional prognostic markers, including proteinuria, eGFR, and the Oxford histologic classification, lack sufficient sensitivity and specificity for early risk stratification and prognosis [2]. Recently, transcriptomics has allowed the exploration of changes in gene expression that initiate and progress important pathways in this disease process. Bulk renal transcriptomics (the traditional use of microarray and RNA-seq) and, more recently, single-cell/single-nucleus RNA sequencing (sc/snRNA-seq) have enabled high-resolution multitissue profiling of cellular states and molecular pathways in IgAN [3]. Notably, these technologies can analyze the heterogeneity in both renal compartments, which are most often affected, such as mesangial cells, podocytes, endothelial cells, and tubular epithelium. Furthermore, these technologies allow for the characterization of infiltrating immune cells to further understand spatial, temporal, and functional disease progression. These opportunities provide important and relevant data to closely examine pathophysiological changes and possible prognostic signatures that were previously inaccessible.

This narrative review aims to summarize recent transcriptomic findings in IgAN, highlight their relevance to disease mechanisms and progression, and evaluate their potential to refine current prognostic and therapeutic strategies for IgAN. In particular, we emphasize how multimodal transcriptomic approaches converge on shared biological pathways and how these insights can be leveraged for precision risk models, an angle not comprehensively addressed in earlier reviews. To move beyond descriptive gene lists, we will (i) distinguish findings replicated across independent cohorts and platforms, (ii) flag signals seen in limited datasets or without outcome linkage, and (iii) explicitly note hypotheses requiring orthogonal and prospective validation in future studies. Although the review is narrative in style, we conducted a methodical examination of the literature to provide a comprehensive search for studies relevant to our review. Using combinations of the following terms: “IgA nephropathy”, “transcriptomics”, “RNA sequencing”, “gene expression”, “single-cell RNA-sequencing”, “spatial transcriptomics”, “urinary transcriptomics”, and “biomarkers”, we identified relevant studies. The review included original, peer-reviewed studies published in English. We included studies that were relevant to transcriptomic analyses in IgAN and those that were relevant to bulk tissue, single-cell, spatial, or liquid biopsy data. Our approach provided a broad and representative synthesis of existing evidence.

## 2. Biological Basis of IgAN and Rationale for Transcriptomic Profiling

### 2.1. Pathophysiological Overview

The “four-hit” hypothesis describes the currently accepted model of IgAN pathogenesis. At the core of IgAN is the production of aberrantly glycosylated immunoglobulin A1, specifically Gd-IgA1. Due to deficient O-glycosylation in the hinge region, Gd-IgA1 becomes immunogenic and triggers the generation of autoantibodies (typically IgG or IgA). Once deposited, immune complexes bind to receptors, resulting in mesangial cell proliferation, extracellular matrix (ECM) accumulation, and inflammation [4,5]. More recent literature has described the upregulation of chemokines and the antagonism of chemokines [C-C motif chemokine ligand 2 (CCL2), C-X-C motif chemokine ligand 2 (CXCL2)], as well as the classical activation of pathways [macrophage-colony stimulating factor (M-CSF), tumor necrosis factor-alpha (TNF-α), interleukin (IL)-6, complement, and ECM remodeling] [6,7]. As tissue damage progresses, tubular epithelial cells exhibit a signature of stress, tissue injury, and profibrotic features, including upregulation of the genes *LCN2* and *HAVCR1*, demonstrating a transition to interstitial fibrosis and progressive renal dysfunction [8]. Finally, glomerulosclerosis and interstitial fibrosis lead to decreased glomerular filtration rate (GFR) and increased proteinuria [9].

### 2.2. Why Renal Tissue Transcriptomics Matters

Histological grading of IgAN using the Oxford classification offers a static snapshot of glomerular architecture, such as mesangial proliferation and interstitial fibrosis. However, it does not capture the active molecular processes. It is important to note that transcriptomic profiling has the potential to enhance risk stratification in the Oxford MEST categories, as it reflects a biological process that occurs before or after the inflammatory lesions described histologically. For example, the molecular markers of tubular stress [hepatitis A virus cellular receptor 1 (HAVCR1) or kidney injury molecule-1 (KIM-1) and lipocalin-2 (LCN2), also known as neutrophil gelatinase-associated lipocalin (NGAL)] tend to be upregulated even in biopsies with little to no interstitial fibrosis and tubular atrophy (IFTA). Likewise, assessing glomerular CCL2 and CXCL10 may aid in delineating inflammatory activity that is not evident from mesangial scores alone. Leveraging transcriptomic indices and related clinicopathologic predictors could improve the predictive metrics of existing risk calculators, such as the International IgAN Prediction Tool.

In contrast, renal tissue transcriptomics (including bulk RNAseq and spatial RNA-seq) provides much more granular details regarding real-time gene expression, which reflects ongoing disease processes [3]. Bulk RNA-seq involves the extraction and sequencing of RNA from either whole sections of tissue or microdissected structural features (e.g., glomeruli or tubulointerstitium) and typically has a multi-step workflow to optimize RNA integrity and capture the entire transcriptome. In most studies, tissue, fresh-frozen, or formalin-fixed paraffin-embedded (FFPE) biopsy samples are used. If compartment-specific resolution is required, either laser capture microdissection (LCM) or manual microdissection under a stereomicroscope can be used to isolate glomeruli or the cortical tubulointerstitial region to diminish transcript “dilution” by other cell types [10,11]. RNA extraction is performed using phenol–chloroform extraction (e.g., TRIzol) or silica-membrane column kits specifically designed for low input amounts and often includes DNase treatment to remove genomic DNA contamination. RNA integrity is evaluated using capillary electrophoresis (for example, Agilent Bioanalyzer) by calculating the RNA integrity number (RIN), as degraded RNA can skew downstream quantification. Library preparation is usually performed by either selecting poly(A) or depleting ribosomal RNA (relevant to degraded FFPE RNA), followed by reverse transcription to cDNA, fragmentation, adapter ligation and PCR amplification. Sequencing is usually performed on concomitant Illumina sequencing platforms, resulting in short-read data at depths of approximately 20–50 million reads per sample to ensure the sufficient detection of low-abundance transcripts [12,13].

Spatial transcriptomics further refines this approach by precisely aligning expression data with histologic compartments. It encompasses a group of cutting-edge methodologies for mapping global gene expression within intact tissue architecture, preserving the essential spatial context of molecular activity. In contrast to bulk or scRNA-seq, these techniques overlay transcriptomic profiles onto histologic images, allowing the identification of spatial gene expression patterns, cellular heterogeneity, and microenvironmental interactions within tissues. Spatial transcriptomics has opened new pathways for constructing detailed molecular atlases of renal structures, understanding disease mechanisms, and improving clinical phenotyping [14]. Platforms such as 10X Genomics Visium (Visium Spatial Gene Expression) enable transcript capture from tissue sections using spatially barcoded oligo-dT spots, aligned with hematoxylin and eosin (H&E) staining for morphological context [15]. This integrated approach has been used effectively in both mouse and human kidney specimens, allowing transcriptome mapping at near single-cell resolution and linking molecular signatures directly to the histopathological landmarks. Importantly, spatial transcriptomics holds promise for revealing disease-specific microenvironments, informing our understanding of acute kidney injury (AKI), chronic kidney disease (CKD) progression, and potential therapeutic targets [16].

**Figure 1:**
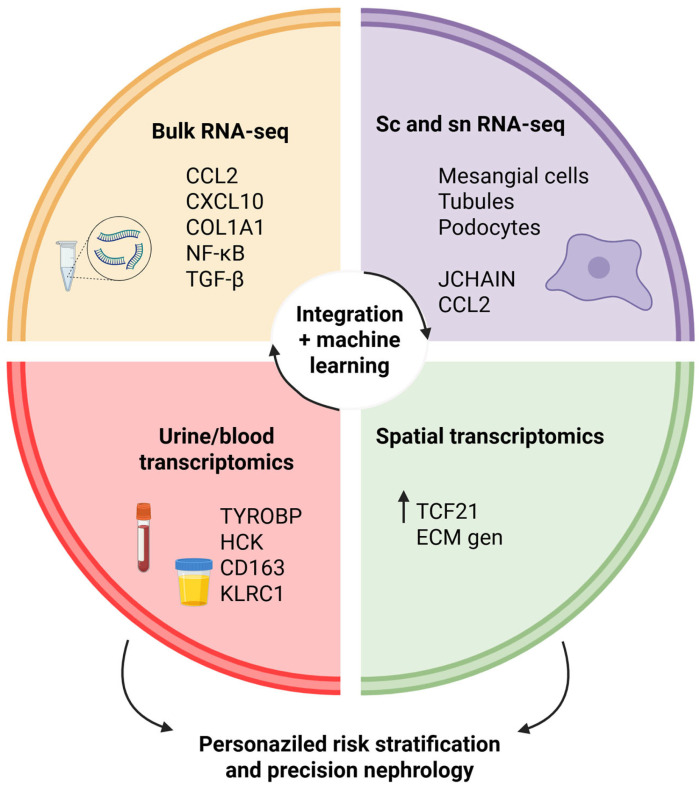
Transcriptomic approaches in IgA nephropathy. Overview of major transcriptomic strategies applied to IgAN. Bulk RNA sequencing provides compartment-specific gene expression profiles (i.e., *CCL2*, *CXCL10*, *COL1A1*), and pathways (e.g., NF-κB and TGF-β). Single-cell and single-nucleus RNA sequencing resolves cell type-specific signatures in mesangial, podocytes, tubular epithelium, and immune cell subsets while concurrently identifying disease-associated genes (e.g., *JCHAIN*, *CCL2*). Spatial transcriptomics maintains tissue architecture and correlates molecular perturbations (e.g., *TCF21*, extracellular matrix genes) with histopathology. Likewise, urine and blood transcriptomics facilitate biomarker discovery (i.e., TYROBP, HCK, CD163, KLRC1) and longitudinal tracking in a non-invasive manner. The integration of these approaches with machine learning produces predictive models that allow for personalized risk stratification and precision nephrology.

scRNA and snRNA-seq offer opportunities to dissect tissue heterogeneity by profiling gene expression at the single-cell or single-nucleus level. Each approach has advanced our understanding of pathophysiologic states [17] and allowed for the identification of unique (e.g., rare or transitional) renal cell types, including previously unknown renal cell types. Unlike scRNA-seq, which requires dissociation into live cells and captures both cytoplasmic and nuclear transcripts, snRNA-seq isolates nuclei, making it particularly suitable for frozen or preserved tissues and reducing dissociation-related transcriptional artifacts. Comparative analyses of adult human kidneys have shown that snRNA-seq identifies rare fibrotic or matrix-embedded cell populations more effectively, achieving gene detection rates comparable to those of scRNA-seq while minimizing bias from difficult-to-dissociate cell types [18]. Furthermore, meta-analyses integrating multiple sc and snRNA-seq datasets from healthy adult kidneys have established consensus transcriptional signatures for 24 distinct renal cell types, including mesangial cells, podocytes, endothelial subtypes, proximal and distal tubular epithelial subsets, collecting duct principal and intercalated cells, and immune cell populations. These standardized reference maps improve reproducibility across platforms and provide a benchmark for cell type annotation in disease-focused studies, thereby facilitating cross-cohort comparisons and mechanistic interpretation of IgAN transcriptomics [19]. An overview of transcriptomic approaches applied to IgAN, ranging from bulk RNA-seq to noninvasive urine and blood transcriptomics, is presented in Figure 1.

## 3. Bulk Transcriptomic Studies in IgAN

Bulk transcriptomic profiling using microarray and RNA seq platforms has greatly advanced our understanding of the molecular mechanisms underlying IgAN. High-throughput approaches can quantify thousands of transcripts from kidney tissue from patients simultaneously and elucidate the molecular programming underlying disease initiation, progression, and clinical heterogeneity. By examining gene expression in microdissected glomeruli and tubulointerstitial compartments, compartment-specific molecular signatures have been used as proxies for histopathological lesions, including mesangial hypercellularity, endocapillary proliferation, and IFTA. Early work using microarray technology provided the first detailed compartment-specific transcriptional map of IgAN. Analysis of microdissected glomeruli revealed the upregulation of ECM components, including perlecan (HSPG2), decorin (DCN), and biglycan (BGN), alongside the activation of the transforming growth factor-beta (TGF-β) pathway, consistent with mesangial matrix expansion and fibrosis [20]. The dataset GSE37460, generated in this early period, has become a reference resource for IgAN research. More recently, Miraji et al. (2019) re-analyzed GSE37460 alongside GSE93798 and GSE104948, identifying the enrichment of pro-inflammatory chemokine genes, such as *CX3CR1* and *CCL4*, as well as complement and ECM-related genes, including *CD44* and *FN1*, in glomerular compartments, supporting a model of coordinated immune activation and matrix remodeling in IgAN [21].

You et al. (2025) [1] reanalyzed the bulk RNA-seq dataset GSE243078 and found significant enrichment of pathways involved in basement membrane regulation, collagen trimer formation, type II interferon signaling, classical complement activation, and focal adhesion in the cells. In their analysis, renal cortical tissue from patients with biopsy-proven IgAN and healthy controls was processed using standardized RNA-seq pipelines, followed by normalization and differential expression analysis using stringent false discovery rate (FDR) thresholds. Enrichment analysis of Gene Ontology (GO) and Kyoto Encyclopedia of Genes and Genomes (KEGG) not only revealed structural and immune-related pathways, but also suggested other pathways related to signaling modules in cytoskeletal organization and leukocyte adhesion, indicating that mechanical–structural changes to the glomerular filtration barrier may occur with immune activation (due to inflammatory injuries in the parenchyma). Interestingly, the measured complement activation signals included both classical (C1QA and C1QB) and alternative pathway (CFB and C3) components, confirming the histological evidence of complement deposition in IgAN. The authors also cross-validated their findings by examining independent microarray datasets, such as GSE37460 and GSE104948, which showed strong reproducibility across patient cohorts and data platforms.

Pathway analyses from these datasets clearly highlighted the activation of nuclear factor kappa-light-chain-enhancer of activated B cells (NF-κB) signaling, the primary transcriptional regulator of inflammation, which is activated upon stimulation of Toll-like receptors (TLRs), cytokine receptors, and immune complexes. NF-κB activation results in the overexpression of target genes (*CCL2*, *CXCL8*, *TNF*, *IL1β*, and *ICAM1*) that mediate leukocyte adhesion, migration, and pro-inflammatory propagation in the glomeruli and tubulointerstitium [1,22]. NF-κB is overexpressed in transcriptomics, and this finding is supported by immunohistochemistry, showing nuclear translocation of NF-κB p65 in mesangial and tubular epithelial cells in IgAN biopsies [23]. TGF-β signaling is the master regulator of fibrogenesis in IgAN. It overexpresses ligands TGFB1 and TGFB2, receptors TGFBR1/2, and transcription factors SMAD and SMAD3, which mechanistically induce transcriptional effects on ECM genes (e.g., *COL1A1*, *COL3A1*, *FN1*, and *SERPINE1*), which then induce mesangial matrix expansion and interstitial fibrosis [1,20]. Elevated urinary TGF-β1 levels have been shown to predict disease progression in IgAN, supporting transcriptomic data indicating active TGF-β pathway involvement. In a large cohort study, both urinary IL-6 and TGF-β1 levels were significantly elevated in IgAN patients compared to those in healthy controls. Although IL-6 was an independent predictor of progression, TGF-β1 levels also correlated with deterioration in kidney function and were associated with IgAN progression before adjustment for clinical factors [24]. The Janus kinase/Signal Transducer and Activator of Transcription (JAK/STAT) signaling pathway, typically linked to cytokine-mediated immune responses, was also significantly stimulated. In IgAN, activation is reflected in the increased expression of STAT1, STAT3, JAK1, and their target genes, many of which are also induced by interferon-γ (e.g., *CXCL9*, *CXCL10*, *GBP1*). This suggests an active interplay between adaptive immune responses and resident renal cells, contributing to mesangial cell proliferation and tubular epithelial dysfunction [1,22]. Although direct experimental IgAN models demonstrating JAK/STAT inhibition efficacy are pending, extensive evidence from diabetic nephropathy models, in which STAT3 pathway blockade reduced proteinuria and histological injury, supports the potential of JAK/STAT targeting. Furthermore, JAK/STAT activation is clearly present in human IgAN tissues, underlining its relevance as a therapeutic target [25,26]. Selective JAK inhibitors, such as baricitinib, tofacitinib, and upadacitinib, have demonstrated anti-proteinuric and anti-inflammatory efficacy in other glomerular or autoimmune diseases, including but not limited to lupus nephritis and diabetic kidney disease. However, no randomized controlled trials have been published assessing their ultimate efficacy in IgAN. Therefore, the targeting of JAK/STAT remains prospective and hypothesis-generating. Future clinical studies will need to assess class-specific safety issues (risk of infection, possible abnormalities of lipid profile, thromboembolic events) and investigate whether blockade of the pathway provides an additive benefit versus standard corticosteroids or as complement-directed agents in IgAN.

In addition to these canonical inflammatory and fibrotic pathways, Park et al. [22] identified the enrichment of chemokine signaling (driven by CCL2 and CXCL10), B-cell receptor activation, Fcγ receptor-mediated phagocytosis, and spleen tyrosine kinase (SYK) signaling in IgAN glomeruli, suggesting robust activation of both innate and adaptive immune receptor pathways. SYK upregulation was validated by immunohistochemistry in kidney tissue and functionally confirmed in cultured mesangial cells, where stimulation with IgA-containing immune complexes induced SYK-dependent pro-inflammatory gene expression, linking transcriptomic signals to functional immune activation in the kidney. The complement cascade is another enriched pathway, in agreement with the histological evidence of mesangial C3 deposition, a diagnostic hallmark of IgAN [27]. Transcriptomic analyses suggest the activation of complement pathways in IgAN renal tissue, although complementary proteomic and histological studies provide stronger direct evidence. Proteomic profiling has revealed a significant accumulation of C3 and C9 in the glomeruli via mass spectrometry, indicative of classical, lectin, and alternative pathway involvement [28]. Immunohistochemical analysis confirmed that patients with IgAN who progressed to kidney failure exhibited heightened glomerular staining for C1q, C3, factor B, C4d, and C5b-9, supporting the contribution of multiple complement factors to disease progression [29].

Bulk transcriptomic signatures not only encapsulate the underlying histopathology but also correlate with disease trajectory. Increased glomerular expression of CCL2, LCN2, and COL1A1 correlated with more significant baseline proteinuria, increased IFTA, and a more rapid eGFR decline. Glomerular RNA profiling at the time of diagnostic biopsy could predict 10-year renal outcomes, even in patients categorized as low-risk pathology only with histology, providing a complementary molecular data component for upfront risk stratification and treatment [30].

Across multiple studies, certain transcripts are consistently upregulated in IgAN renal tissue. For example, monocyte chemoattractant protein-1 (CCL2) was robustly elevated in the glomerular and tubulointerstitial compartments in the RNA-seq study by Park et al. [22] and confirmed in the microarray dataset GSE37460 [9], correlating with macrophage infiltration scores and proteinuria. CXCL10, another chemokine, was upregulated [1,22], which aligned with the enrichment of type II interferon signaling pathways. COL1A1, a marker of active ECM deposition and fibrosis, was found to be significantly overexpressed in the study byf You et al. [1] and in GSE104948, with expression levels correlating with Oxford T scores and interstitial fibrosis severity. NGAL was markedly increased in the tubulointerstitial compartment [22,31], where it correlated with histologic injury and eGFR decline. KIM-1 expression was similarly elevated in the tubulointerstitial compartment [22] and detected at high levels in urine RNA-seq [32], both indicating tubular epithelial stress and dedifferentiation.

Integrated computational analyses of bulk transcriptomic data have greatly increased our knowledge of the underlying molecular processes of IgAN. For example, genome-wide weighted gene co-expression network analysis (WGCNA) can be used on microarray datasets. The microarray datasets GSE93798 and GSE115857 were used to create modules for correlated gene expression signatures. Ren et al. (2024) used WGCNA to create three functional IgAN subtypes (“viral-hormonal”, “bacterial-immune”, and “mixed”) with subtype-specific modules that enriched inflammatory and immune infiltration, effectively identifying the diverse pathogenic processes across IgAN patients [33]. Beyond WGCNA, targeted analyses of necroptosis have identified key necroptosis-related differentially expressed genes in IgAN. Hu et al. (2024) [34] cross-analyzed microarray datasets (GSE93798 and GSE115857) with curated necroptosis gene lists, identifying seven “hub” necroptosis-related genes, including *JUN*, *NFKBIA*, and *CD274*, through LASSO regression and validation in an scRNA-seq dataset (GSE171314). This study underscores the role of necroptosis and inflammatory signaling as potentially vital contributors to IgAN progression.

Overall, these integrative approaches shed light on the networks of immune response, ECM organization, oxidative stress, and necroptosis in the pathogenesis of IgAN. Selected findings from bulk transcriptomic studies on IgAN are summarized in Table 1.

The majority of reproducible signals across both microarray and RNA-seq studies, as well as in both serum and urine studies, are related to NF-κB/TGF-β/complement pathway enrichment and increased CCL2, CXCL10, LCN2, HAVCR1, and COL1A1, which are associated with proteinuria, IFTA, and eGFR decline. Activation of the JAK/STAT signaling pathway has been consistently found in microarray studies. However, there are limited IgAN-specific tissue studies that support these findings, and even fewer interventional studies have been conducted. Necroptosis-related hubs identified by secondary bioinformatics (e.g., JUN, NFKBIA, and CD274) are hypothesis-generating and require protein-level and functional validation in independent biopsies. Given the reuse of historical GEO cohorts, we caution against circular replication. Future work should pre-register analysis plans, include multi-ethnic cohorts, and confirm RNA signatures with orthogonal assays (IHC/ELISA) and predefined clinical endpoints.

In conclusion, these investigations highlight the concordance of immune activation, ECM remodeling, and complement dysregulation across independent cohorts. Instead of treating each dataset separately, we suggest that the synthesis illustrates that bulk transcriptomics collapses into a reproducible set of pathways (NF-κB, TGF-β, JAK/STAT, and complement) that together drive IgAN progression. Additionally, this integrated perspective clarifies that transcriptomic signatures can provide mechanistic anchors for novel therapeutic strategies.

## 4. Single-Cell Transcriptomics in IgA Nephropathy

While bulk transcriptomics has provided a foundational understanding of gene expression in IgAN, it inherently averages signals across cell types, masking the heterogeneity that defines this complex disease state. The emergence of scRNA-seq and snRNA-seq technologies has revolutionized the ability to resolve transcriptomic landscapes at the level of individual cells. One of the first scRNA-seq studies in IgAN was conducted by Zheng et al. [35]. This landmark study profiled over 23,000 single cells obtained from kidney biopsies and CD14^+^ peripheral blood mononuclear cells (PBMCs) from both IgAN patients and healthy controls. In glomerular tissue, mesangial cells in IgAN uniquely overexpress *JCHAIN*, which encodes the immunoglobulin J chain. This finding suggests that mesangial cells may directly contribute to the in situ formation or retention of polymeric IgA1 complexes, offering a potential mechanistic basis for mesangial IgA deposition. Beyond mesangial cells, a broader inflammatory and fibrotic landscape exists across multiple renal parenchymal cell types. A transitional cell population was identified among intercalated cells exhibiting profibrotic transcriptomic signatures that aligned with early interstitial fibrosis pathways. Additionally, multiple epithelial lineages, including tubular cells, showed aberrant activation of pro-inflammatory pathways, notably IL-17 and TNF signaling, suggesting a widespread injury response that extended beyond the glomerulus. The study also characterized aberrant cellular crosstalk, demonstrating that mesangial cells in IgAN have increased communication with immune infiltrates and neighboring renal cells, supporting the evidence of mesangial-associated multicellular inflammation. Furthermore, the PBMC data provided single-cell immune landscapes with activation patterns in peripheral immune signatures, including the dominance of certain monocyte and T cell populations, which paralleled intrarenal inflammatory changes. Building on these findings, a cross-species scRNA-seq study by Chen et al. [7] compared human IgAN kidneys with those of a mouse model and uncovered conserved molecular signatures. They identified mesangial cells with CXCL12, CCL2, and CSF1 overexpression, suggesting an inflammatory phenotype that orchestrates macrophage recruitment through ligand–receptor interactions, such as CXCL12/CXCR4 and CSF1/CSF1R. Notably, the recruited macrophage subpopulations showed enrichment in antigen presentation and cytokine production programs, consistent with chronic inflammation and fibrotic remodeling.

In a pivotal snRNA-seq study, You et al. (2025) profiled kidney biopsy specimens representing the full spectrum of IgAN, from early stages with preserved kidney function to late-stage disease with significant eGFR decline, integrating their data with those of healthy controls to construct a high-resolution cellular atlas [1]. Among the various cell types annotated, two mesenchymal stromal cell (MSC) clusters exhibited significantly enhanced transcriptomic signatures associated with complement activation, focal adhesion, and collagen formation compared to other renal compartments. Notably, these perturbed MSC clusters became more transcriptionally dominant as eGFR worsened, suggesting their potential role in driving CKD progression. Using pseudotemporal trajectory analysis (e.g., Monocle3 or VIA algorithm), a dynamic transition of mesangial cells towards a myofibroblast-like phenotype was followed, as indicated by the progressive upregulation of functionally similar biological processes related to ECM assembly, components of the complement pathway, effectors of humoral immunity, and structural adhesion molecules. Importantly, in situ knockdown of transcription factors, including Paired Related Homeobox 1 (PRRX1), partially reverted the progression of myofibroblast differentiation. The transformation of mesangial cells was also correlated with the transcriptional trajectories of podocytes and tubular epithelial cells, which increasingly expressed genes characteristic of epithelial-to-mesenchymal transition, hypoxia response, and pathways related to cellular injury, highlighting their involvement in the evolving fibrotic microenvironment.

Complementing the scRNA-seq and snRNA-seq studies, Park et al. [6] utilized the GeoMx Digital Spatial Profiler to conduct high-resolution spatial transcriptomic profiling on kidney biopsy sections from IgAN patients at different stages of mesangial proliferation, categorized according to the Oxford M-score (M1 vs. M0), as well as matched donor controls. Multiple glomeruli per biopsy were selected and precisely annotated into regions of interest (ROIs) corresponding to known histopathological variations. Their analysis revealed a set of 77 upregulated and 55 downregulated differentially expressed genes (DEGs) distinguishing M1-IgAN from control glomeruli, with additional DEGs identified between M1 and M0 IgAN, as well as between M0 and controls. Notably, epicardin (TCF21), a marker of early podocyte injury, was the only DEG consistently elevated in both M1 and M0 IgAN groups compared to the controls. Key transcription factors, such as *ATF3*, *EGR1*, *DUSP1*, *FOS*, *JUNB*, *KLF2*, *NR4A1*, *RHOB*, and *ZFP36*, were uniformly downregulated across IgAN samples. Functionally, glomeruli exhibiting mesangial proliferation (M1) were enriched in cell surface adhesion molecules, vascular development genes, and ECM components, reflecting localized structural changes. This spatial resolution allowed them to directly link transcriptomic changes to specific histologic architecture, showing that early mesangial activation co-occurs with structural remodeling and possibly inflammation in the adjacent periglomerular areas. An overview of single-cell and spatial transcriptomic discoveries in IgAN is presented in Table 2.

Analyses should anchor batch correction to consensus kidney cell atlases and report cross-study cell type agreement to ensure portability. The cellular atlas of IgAN has been further refined by recent single-cell studies. In addition to the spatial endothelial–mesangial findings established by Hasegawa et al. [36], Ye et al. [37] used the same scRNA-seq approach in children with IgAN and IgA-vasculitis-associated nephritis to corroborate that while there were age-related differences in disease characteristics, the same mesangial–immune activation pathways were engaged, regardless of age. These complementary approaches underscore the possibility that the same disease mechanism measured in adult IgAN may operate earlier in life and justify transcriptomic work with stratified analyses based on age.

## 5. Urinary and Blood Transcriptomics as Non-Invasive Biomarker Platforms

Beyond biopsy-based studies, urinary bulk RNA-seq has emerged as a promising non-invasive tool for transcriptomic profiling in IgAN, offering the ability to monitor disease activity without repeated invasive sampling. This approach capitalizes on the fact that urine contains shed renal epithelial cells, leukocytes, and extracellular vesicles (including exosomes and microvesicles) originating from the kidney and urinary tract, which preserve mRNA transcripts that are reflective of in situ renal biology. Urinary sediment can be processed directly after centrifugation, or RNA can be isolated from exosome-enriched fractions using ultracentrifugation, precipitation or size-exclusion chromatography. Xie et al. [32] sequenced RNA from urinary sediment and exosomes collected from 53 IgAN patients and matched healthy controls. They employed both poly(A)-selected and ribosomal RNA–depleted library preparation strategies to maximize transcript detection, achieving a median sequencing depth of ~30 million paired-end reads per sample. Using machine learning classifiers (random forest and LASSO regression), they identified TYRO protein tyrosine kinase-binding protein (TYROBP) and hematopoietic cell kinase (HCK) as highly predictive biomarkers of IgAN, with expression levels correlating with estimated glomerular filtration rate (eGFR) decline and serum creatinine levels over longitudinal follow-up. Immunohistochemistry confirmed that both TYROBP and HCK were upregulated in diseased kidney tissue, predominantly in infiltrating macrophages and activated mesangial cells, linking urinary transcriptomic changes to intrarenal immune activation. Urinary MCP-1 (CCL2) levels were consistently correlated with the clinical markers of disease severity in IgAN. For example, Saitoh et al. [38] found that urinary MCP-1 levels were significantly higher in advanced IgAN, correlating with the presence of urinary casts and proteinuria. Elevated urinary exosomal *CCL2* mRNA levels have also been linked to active histologic injury and reduced kidney function [39], reinforcing the concept that urinary transcriptomic shifts reflect intrarenal inflammation. In addition to chemokine transcripts, several urinary mRNAs encoding injury and immune activation markers have been validated in independent IgAN cohorts. For example, urinary CD163 mRNA, a marker of alternatively activated macrophages, correlated strongly with histologic tubulointerstitial inflammation and proteinuria severity, supporting its role as a noninvasive indicator of macrophage-driven injury [40]. Kim et al. [31] took the urinary transcriptomic approach one step further with the identification of elevated IL-6, CD14, DNA (Cytosine-5)-Methyltransferase 1 (DNMT1), FK506 Binding Protein 5 (FKBP5), and nephrin (NPHS1) in IgAN patients, with several correlating significantly with Oxford MEST scores (especially M and T lesions) and with longitudinal eGFR decline over a median follow-up of 4.5 years. The detection of podocyte-associated transcripts (i.e., nephrin) may indicate that urinary RNA-seq has a role in identifying actively damaged podocytes, and the detection of DNMT1 and FKBP5 implicates epigenetic control and stress response programs in a continuously progressive disorder. Overall, these studies establish that urinary transcriptomic signatures (either urine sediment or extracellular vesicle fractions) can capture active renal pathology, correlate with histology and functional evidence of the underlying disorder, and offer prognostic information.

Although these results are encouraging, their clinical use is still in its infancy. The predictive models proposed so far, urine TYROBP/HCK or CCL2/CD163 signatures, have shown cross-cohort reproducibility, but their external validity has been challenged. While the reported discrimination (AUC ≈ 0.80–0.88) is reasonable, it is rare to assess the calibration and performance of clinical reclassification models. Urinary CCL2/MCP-1 and CD163 mRNA are associated with activity and histology across studies and are closest to pragmatic monitoring biomarkers, pending assay standardization and cut-points. Urinary TYROBP/HCK machine-learning panels show promise but require prospective, multi-site validation with locked models and head-to-head comparisons with established markers. Classifiers based on PBMCs (e.g., KLRC1 and C1QB) carry the risk of confounding due to intercurrent infections complicated by systemic inflammation. Future investigations should incorporate negative controls (e.g., other glomerulonephritides and viral diseases), preanalytical quality assurance, and blinded adjudication of outcomes.

To facilitate clinical translatability, further studies should include locked external validation and prospective multicenter cohorts with standardized assay protocols. Concrete next steps to accomplish this include harmonizing RNA isolation and normalization methods, instrumenting homogeneous clinical endpoints, and embedding transcriptomic assays into existing IgAN registries and therapeutic trials. These steps will work toward closing the translational gap and provide the evidence base needed for reliable and routine clinical use. Starting in 2023, several studies have improved the urinary transcriptomic and exosomal biomarker fields. Yoon et al. [41] reported on a group of urinary exosomal microRNA that distinguished IgAN from healthy controls with an AUC of over 0.90. Similarly, Shankar et al. [42] described a urinary exosomal miRNA signature that correlated with histologic activity and proteinuria. In a more recent study, Zhao et al. [43] determined that urinary exosomal mRNAs could predict how patients would respond to treatment with renin–angiotensin system (RAAS) inhibitors, linking transcriptomic readouts to therapeutic efficacy. Collectively, these studies are not solely focused on biomarker discovery but mark a transition towards clinically informative, treatment-responsive transcriptomic assays. The representative urinary and blood transcriptomic biomarkers of IgAN are summarized in Table 3.

Concurrent advances in blood transcriptomics have added new dimensions to our understanding of systemic immunodysregulation in IgAN. Bulk and scRNA profiling of systemic PBMCs has revealed interesting findings on systemic transcriptional programs that reflect intrarenal inflammatory signatures. For example, a bulk RNA-seq study [44] examined PBMCs of IgAN patients and healthy controls and reported 333 differentially expressed genes from RNA-seq in IgAN patients enriched in NK cell-mediated cytotoxicity, complement activation, antigen processing, and B-cell receptor (BCR) signaling pathways. Two hub genes, *KLRC1* and *C1QB*, exhibited high diagnostic accuracy and were notably correlated with proteinuria and eGFR decline. Zeng et al. (2021) [45] identified immune subsets related to disease on a single-cell basis, highlighting interferon-responsive monocytes, atypical B cell populations, and shifts in gene expression patterns, all characterized relative to clinical severity. These studies provide evidence that blood-based transcriptomics is a minimally invasive platform for characterizing immune cues to identify disease progression and, more importantly, potentially stratify therapies for IgAN.

Together, these urinary and blood transcriptomic studies show that noninvasive signatures mirror intrarenal pathology, enabling real-time disease monitoring. Integrating tissue-based and liquid biopsy transcriptomics may provide the most robust framework for precision risk stratification, surpassing the limitations of biopsy alone.

## 6. Comparative Insights Across Transcriptomic Approaches

Each transcriptomic modality presents a unique perspective on IgAN, and a complementary perspective is produced by directly comparing the modalities to one another. Bulk transcriptomics (microarray or RNA-seq) remains the workhorse for transcriptome-wide discovery, allowing the investigation of altered pathways, including NF-κB, TGF-β, JAK/STAT, and complement activation, and correlations to clinical outcomes such as proteinuria or eGFR decline prove insightful. Its rigor and reproducibility across cohorts, as well as the ease of utilizing archived specimens, provide a sensible starting point. Nevertheless, averaging across various complex cell types does not allow for robust cell-type-specificity attributions and may overlook important heterogeneity. Table 4 provides a systematic high-level summary of the advantages, disadvantages, and applications of each approach.

scRNA-seq and snRNA-seq profile transcriptomes at the individual cell or nucleus level circumvent this restriction. These techniques have characterized mesangial and stromal populations as key contributors to inflammation and fibrosis in IgAN, identified pro-inflammatory tubular and mesangial states, and elucidated immunological crosstalk networks in the peripheral and renal compartments. For example, a recent review emphasized how scRNA-seq is advancing our mechanistic understanding and biomarker discovery in IgAN [46]. snRNA-seq is especially useful for frozen or archived tissue, mitigating dissociation bias. Nevertheless, these high-resolution methods require substantial resources, rigorous batch correction, and careful interpretation in small cohorts.

Spatial transcriptomics occupies an intermediate niche by preserving tissue architecture and mapping molecular signatures onto histological regions. In IgAN, a recent study using GeoMx Digital Spatial Profiling compared glomeruli with mesangial proliferation (M1) versus non-proliferative (M0) and controls, identifying *TCF21* as a consistently upregulated gene and implicating adhesion and ECM pathways in proliferative lesions [6]. This demonstrates how spatial mapping can reveal structure-specific molecular shifts. In other glomerular diseases, spatial transcriptomics has shown that even histologically similar glomeruli can harbor divergent molecular programs, reinforcing the value of the spatial context [47]. However, current spatial platforms often have limited resolution (spots capturing multiple cells), higher costs, and lower throughput, which limits their scalability. 

Liquid biopsy transcriptomics (urine and blood) offers the most direct clinical translation pathways. In IgAN, urinary RNA-seq studies have nominated TYROBP and HCK as non-invasive biomarkers with machine-learning validation across cohorts, correlating with renal function decline [32]. Another study of urinary biomarkers (in CKD) demonstrated that mRNAs, lncRNAs, and circRNAs in urine can reflect renal injury processes [48]. In addition, urinary osteopontin (SPP1) has been proposed as a “kidney–urine-linked” biomarker using scRNA-seq data for cross-validation [49]. These liquid biopsy signals allow repeated sampling over time, providing access to dynamic disease monitoring. However, they can be confounded by systemic processes or variable RNA yields and must be validated against tissue-derived signatures to determine tissue specificity.

These modalities should be interpreted as layer upon layer of insight, working together rather than competing. Bulk approaches define strong pathway-level perturbations; single-cell and single-nucleus sequencing map those signals into specific cell types and states; spatial transcriptomics frame those cell types and states within their histological microenvironments; and liquid biopsy makes all of this material meaningful for the clinic through a longitudinal accessible marker. An integrated strategy, including bulk discovery, refined by cellular resolution, spatially anchored, and carried forward via noninvasive biomarkers, offers the most comprehensive path toward mechanistic insight and precision nephrology in IgAN.

## 7. Challenges and Limitations

Transcriptomic tools may transform IgAN management; however, significant hurdles remain before they can benefit patient care. There are limits to the clinical translation of transcriptional modalities. These limits cover the technical, clinical, and ethical domains that demonstrate the complex convergence of omic science, patient care, and data sensibility. From a technical perspective, the quality and preservation method of renal tissue are critical determinants of transcriptomic data quality. While fresh-frozen tissue is preferred to ensure RNA integrity, it may not be easily used in specialized research protocols. In clinical nephrology, FFPE specimens are routinely obtained. However, RNA extracted from FFPE tissue is often degraded and/or chemically modified, and the extracted RNA may introduce noise in complementary gene expression analyses. Although some developments in library preparation protocols for FFPE tissue have been made to improve performance, they are limited in terms of maximal transcript coverage or depth. This situation may be further complicated by analytical limitations due to batch effects that could occur when the samples are handled, when RNA is extracted, and at the time of sequencing, which is further complicated when data from different patients, sites, or platforms are combined [6,36,50,51].

In addition to these logistical and technical issues, several analytical challenges exist in transcriptomic studies. Additionally, some biological heterogeneity (i.e., patient age, medications, comorbidities, or circadian variation) can also contribute to variability in the expression profile and mask biologically significant signals related to the disease. Related to this, and of concern, is the issue of statistical overfitting, where studies collectively utilize high-dimensional data but with limited numbers of samples, leading models to capture noise rather than true biological variance. Cross-validation, external replication, and applying model regularization (e.g., LASSO or elastic net) will all be important to overcome this. Furthermore, sample handling and storage conditions contribute significantly to RNA integrity, and inconsistent handling before analysis can introduce spurious differential expression. Moving forward, to address these variability and bias issues, we will seek to improve reproducibility through transparent reporting of processing, sharing of codes and raw data, and benchmarking against a common reference dataset. More importantly, if studies are not statistically corrected sufficiently for possible variability or lack sufficiently matched comparative groups for those transcriptomic signatures, the findings are unlikely to be generalizable or reproducible in other independent cohorts. Most transcriptomics studies to date have been either exploratory or retrospective, often limited by cohort size and short-term follow-up periods. This has resulted in a lack of prospective validation of gene signatures and many candidate markers without established prognostic significance.

In addition, reproducibility across cohorts and sequencing platforms remains a key limitation. Many IgAN datasets rely on historical GEO series with small, ethnically homogeneous populations, and few studies have applied standardized normalization pipelines across RNA-seq, microarray, and spatial platforms. Harmonization efforts, such as those from the Kidney Precision Medicine Project and Human Cell Atlas Kidney Initiative, should be expanded to enable true cross-study benchmarking and meta-analytic reproducibility. The publication of large-scale multimodal atlases, such as that of Lake et al. [52], provides a valuable benchmark for quality control and annotation consistency. The integration of IgAN datasets with standard references is essential to reduce batch effects and ensure reproducibility across platforms. Cost and scalability are major impediments to clinical adoption. Comprehensive single-cell or spatial assays continue to be prohibitively expensive, costing more than 1000 USD per assay, largely because they utilize specialized imaging, sequencing depth, and bioinformatics. Therefore, for any future large-scale screening or monitoring application, scalable bulk RNA-seq or targeted multiplex assays are required to cost-effectively and efficiently match clinical practice. Investments in automated analysis pipelines or cloud-based processing may reduce per-assay costs and operator dependency.

To contextualize the current state of translation, transcriptomic biomarkers in IgAN can broadly be categorized by readiness level: (i) exploratory: gene panels such as necroptosis, or PRRX1-related modules identified in single datasets; (ii) validated: urinary CCL2/MCP-1, CD163, and tissue-derived LCN2 or HAVCR1, which have been replicated across independent cohorts with clear correlations to histopathology and renal outcomes; and (iii) near clinical use: machine learning-derived urinary TYROBP/HCK signatures that have undergone multi-cohort testing and show potential as real-time, non-invasive surrogates for disease activity. However, no transcriptomic biomarker has achieved full clinical qualification for regulatory or routine diagnostic use in nephrology [3,6,53].

Another barrier is ethical data sharing. High-quality transcriptomic investigations depend on the ability to pool large and diverse datasets from institutions and geographic boundaries. However, regulatory limitations, data protection laws, and numerous ethical frameworks inhibit the sharing of patient-oriented omics data. Although datasets are presumably available, the metadata footprint, including clinical context data, treatment history, and histologic data, is often missing or inconsistent in how it was annotated, making it difficult to aggregate cross-cohort data. The Kidney Precision Medicine Project (KPMP) is attempting to address some of these limitations by establishing data standards and centralized data coordinating centers; however, widespread adoption is still underway [52,54].

## 8. Future Perspectives

The future of transcriptomic studies in IgAN is to improve both the spatial resolution and temporal granularity of gene expression data while incorporating this molecular information into clinical practice. However, there is very little clinical translation at this time, and calibration should be approached with caution until future multicenter validation is performed to establish its analytical robustness and clinical value. Translational efforts from transcriptomic data in clinical practice will require stepwise clinical validation of transcriptomic assays, similarly to the testing performed in oncology for companion diagnostics. Specifically, biomarkers will help move from their initial discovery (analytical feasibility and biological plausibility) to validation (multi-cohort reproducibility and standardization pipelines). Validation culminates in qualification (prospective clinical utility demonstrated in intervention trials). A major future direction is the integration of transcriptomic readouts directly into existing prognostic frameworks. Models combining Oxford MEST scores, baseline eGFR, and proteinuria with transcriptomic activity indices have already demonstrated improved discrimination for renal outcome prediction in pilot studies. As these tools mature, transcriptomic data could serve as a ‘molecular layer’ within clinical risk calculators, augmenting rather than replacing established histologic and clinical predictors. This integration will likely require standardized bioinformatics pipelines and validation in multicenter cohorts to ensure reproducibility and clinical interpretability. To date, only a few transcriptomic models have been externally validated. Urinary TYROBP/HCK classifiers reported cross-cohort AUCs of 0.83–0.88 for discriminating progressive IgAN, whereas urinary CCL2 and CD163 mRNA levels have been validated in independent studies with a consistent correlation to eGFR decline and histologic inflammation. However, none of these have been prospectively tested for calibration or clinical reclassification.

One of the most transformative frontiers is spatial transcriptomics, which preserves the anatomical context and localizes disease-relevant gene expression patterns within discrete renal compartments. This approach is particularly valuable in IgAN, where pathological changes are unevenly distributed across the glomeruli, tubulointerstitium, and immune-infiltrated regions. Platforms such as Slide-seqV2 and Visium offer spatially resolved transcriptomic mapping with near single-cell resolution in kidney tissues [55]. Moreover, integrating spatial data with single-cell transcriptomics enhances our ability to pinpoint cell-type-specific gene signatures within histopathological landmarks, enabling insights into disease-related microenvironments in AKI and potentially IgAN [56]. Emerging tools, such as DeepSpot, a deep learning model trained to infer spatial gene expression directly from H&E images, promise to broaden accessibility by transforming widely available histological slides into transcriptomic maps with high spatial fidelity [57].

Another critical direction is the adoption of longitudinal sampling strategies using noninvasive or minimally invasive methods, such as serial urinary transcriptomics. These approaches allow repeated profiling of gene expression over time, enabling real-time monitoring of disease activity, therapeutic response, and risk of progression, which is not feasible with single-timepoint biopsies. This temporal tracking is expected to be instrumental in understanding the dynamic nature of IgAN and facilitating early intervention [3,58,59]. Among the existing candidates, urinary MCP-1/CCL2 and CD163 mRNA have reached a “validated” stage and could feasibly enter multi-center prospective studies to define prognostic thresholds. TYROBP/HCK and composite urinary transcriptomic panels are in an “advanced exploratory” phase with multi-cohort reproducibility but still lack standardized normalization and regulatory-grade assay development. Blood-derived KLRC1/C1QB and interferon-response signatures remain “early exploratory” with minimal external validation. Moreover, transcriptomic biomarkers are being conceptualized as companion diagnostics in clinical studies of novel IgAN therapies. As novel precision therapeutics are being developed at an accelerated pace (including a new generation of therapeutics targeting complement activation, mucosal immunity, and fibrosis), transcriptomic profiles can serve as molecular classifiers for patient subgroup selection, treatment response prediction, and monitoring drug efficacy [60].

To bridge this translational gap, future studies should focus on multicenter prospective cohorts with harmonized protocols for RNA isolation, normalization techniques, and endpoint definition. Multicenter consortia can engage in standard operating protocols for specimen handling and reporting similar to the KPMP. Furthermore, prospective validation using locked prediction models, orthogonal protein-level confirmation, and integration into decision-curve analysis will be essential to demonstrate the added value over established metrics such as eGFR, proteinuria, and the Oxford MEST classification.

Finally, the possibilities created by these advances open the door to a patient-specific treatment model for IgAN, with therapeutic strategies determined rationally based on each patient’s transcriptomic risk signatures. Future diagnostic models that determine which individuals may benefit most from immunosuppression, RAAS blockade, or novel biologics using bulk and scRNA-seq data that correlate with spatial and non-invasive data have the potential to benefit individual patients and open a new era of precision nephrology that is mechanistically driven and prognostically relevant.

## 9. Conclusions

In the past 10 years, transcriptomics has revolutionized our understanding of IgAN and provided a multifaceted molecular snapshot that cannot be characterized solely by histopathology. Transcriptomics, using both bulk and single-cell methods, has revealed that IgAN is a heterogeneous disease, identifying disease pathways, cell-type-specific signatures, and regulatory networks involved in immune activation, fibrosis, and tissue injury, all of which can be explained at the molecular level.

Transcriptomic profiling of renal tissues, notably glomerular and tubulointerstitial tissues, has provided high-resolution, real-time disease activity and dynamic pathophysiological snapshots of the disease. Tissue transcriptomic signatures have shown a strong correlation with clinical parameters (i.e., proteinuria) and disease severity (i.e., histological severity and eGFR decline), supporting their potential application in clinical risk- stratification. Additionally, the capacity of scRNA analysis to pinpoint rare but clinically relevant cell populations and the emergence of non-invasively obtained readouts of tissue-related conditions, such as urinary mRNA profiling, could also serve as a technology that may contribute to potential new biomarkers.

While transcriptomic investigations display potential, there is still very little advantage to using them in clinical decision-making, and they require careful consideration. Current biomarkers should be regarded as adjunctive tests rather than diagnostic tools until multicenter prospective trials have been established to examine discrimination (AUC), calibration, and net reclassification improvement. The integration of molecular data with histological and clinical data will afford clinicians the ability to predict disease trajectories, personalize treatment options and monitor treatment effectiveness. The development of high-quality, validated transcriptomic biomarkers and their integration into prospective trials will be important for eliminating translational gaps.

Currently, transcriptomic biomarkers for IgAN range from exploratory discovery to early clinical readiness. Of the urinary markers studied in IgAN, CCL2 and CD163 were the most mature. Other candidates of interest are still in the proof-of-concept or validation stages. One of the key initiatives will be consensus pipelines, quality standards, and clinical endpoints to operationalize these tools from bench to bedside.

## Figures and Tables

**Table 1:** Bulk transcriptomic findings in IgA nephropathy.

Transcriptomic Compartment	Key Findings	Refs.
Glomeruli	Upregulation of ECM genes (*HSPG2*, *DCN*, *BGN*), TGF-β pathway activation, complement and immune receptor signaling (CCL2, CXCL10, CD44, FN1)	[20,21,22,23,24]
Tubulointerstitium	↑ Injury/fibrosis markers (LCN2, HAVCR1), NF-κB, JAK/STAT activation, necroptosis-related genes (*JUN*, *NFKBIA*, *CD274*)	[22,31,33,34]
Pathway analyses	NF-κB, TGF-β, JAK/STAT, and complement as recurrent enriched pathways	[1,22,25,26,27,29]
Clinical correlations	Higher CCL2, LCN2, and COL1A1 expression linked to proteinuria, IFTA, and faster eGFR decline	[30]

Abbreviations: ECM, Extracellular matrix; HSPG2, Heparan sulfate proteoglycan 2 (perlecan); DCN, Decorin; BGN, Biglycan; TGF-β, Transforming growth factor-beta; CCL2, C-C motif chemokine ligand 2 (monocyte chemoattractant protein-1, MCP-1); CXCL10, C-X-C motif chemokine ligand 10 (interferon gamma-induced protein 10, IP-10); CD44, Cluster of differentiation 44 (cell-surface glycoprotein); FN1, Fibronectin 1; JUN, Jun proto-oncogene, AP-1 transcription factor subunit; LCN2, Lipocalin-2 (neutrophil gelatinase-associated lipocalin, NGAL); HAVCR1, Hepatitis A virus cellular receptor 1 (Kidney injury molecule-1, KIM-1); NF-κB, Nuclear factor kappa-light-chain-enhancer of activated B cells; NFKBIA, Nuclear factor of kappa light polypeptide gene enhancer in B-cells inhibitor, alpha; JAK/STAT, Janus kinase/Signal transducer and activator of transcription; IFTA, Interstitial fibrosis and tubular atrophy; eGFR, Estimated glomerular filtration rate. ↑, increased expression.

**Table 2:** Single-cell and spatial transcriptomic insights in IgA nephropathy.

Approach	Main Discoveries	Refs.
scRNA-seq	Mesangial overexpression of JCHAIN; inflammatory mesangial phenotypes (CXCL12, CCL2, CSF1); EMT signatures in tubules; immune cell crosstalk	[7,35]
snRNA-seq	Mesenchymal stromal clusters enriched for complement activation, collagen formation, and trajectory toward myofibroblast-like states (*PRRX1* as regulator)	[1]
Spatial transcriptomics	M1 glomeruli show ↑ *TCF21* and *ECM* genes; enrichment for adhesion/vascular genes in mesangial proliferation	[7]

Abbreviations: scRNA-seq, Single-cell RNA sequencing; snRNA-seq, Single-nucleus RNA sequencing; JCHAIN, Joining chain of multimeric IgA and IgM; CXCL12, C-X-C motif chemokine ligand 12 (stromal cell-derived factor 1, SDF-1); CCL2, C-C motif chemokine ligand 2 (monocyte chemoattractant protein-1, MCP-1); CSF1, Colony-stimulating factor 1 (macrophage colony-stimulating factor, M-CSF); PRRX1, Paired related homeobox 1 (transcription factor); TCF21, Transcription factor 21 (Pod-1, epicardin); ECM, Extracellular matrix; EMT, Epithelial-to-mesenchymal transition; ↑, increased expression.

**Table 3:** Urinary and blood transcriptomic biomarkers.

Sample Type	Biomarkers Identified	Clinical Relevance	Refs.
Urine (sediment and exosomes	TYROBP, HCK, CCL2, CD163, NPHS1, IL-6, DNMT1, FKBP5	Predictive of eGFR decline, proteinuria, tubulointerstitial inflammation; a surrogate for biopsy	[31,32,38,39,40]
Blood (PBMCs)	KLRC1, C1QB, interferon-responsive monocytes, altered B-cell subsets	Diagnostic accuracy; correlated with proteinuria and eGFR decline	[44]

Abbreviations: TYROBP, TYRO protein tyrosine kinase-binding protein; HCK, Hematopoietic cell kinase; CCL2, C-C motif chemokine ligand 2 (also known as MCP-1, monocyte chemoattractant protein-1); CD163, Cluster of differentiation 163 (hemoglobin–haptoglobin scavenger receptor); NPHS1, Nephrin (podocyte slit diaphragm protein); IL-6, Interleukin-6; DNMT1, DNA methyltransferase 1; FKBP5, FK506 binding protein 5; KLRC1, Killer cell lectin-like receptor C1 (NKG2A); C1QB, Complement component 1, q subcomponent, B chain; PBMCs, Peripheral blood mononuclear cells.

**Table 4:** Comparative overview of transcriptomic approaches in IgA nephropathy.

Approach	Resolution/Scale	Sample Type & Requirements	Key Advantages	Major Limitations
Bulk Transcriptomics (Microarray, Bulk RNA-seq)	Average of thousands of cells per sample	Fresh or archived renal biopsy tissue; compatible with FFPE	- High reproducibility and robustness across cohorts - Enables pathway-level and systems-level analyses - Cost-effective and scalable for large cohorts	- Masks cell-type–specific variation - Cannot resolve cellular heterogeneity or spatial context
Single-Cell RNA-seq (scRNA-seq)/Single-Nucleus RNA-seq (snRNA-seq)	Single-cell/single-nucleus resolution	Fresh (scRNA-seq) or frozen (snRNA-seq) tissue; requires viable cell or nucleus isolation	- Resolves cell-type and state-specific expression profiles - Reveals cellular heterogeneity and crosstalk networks - Enables cell trajectory and pseudotime analyses	- High cost and technical complexity - Sensitive to batch effects and dissociation bias - Limited tissue availability in clinical nephrology
Spatial Transcriptomics (e.g., Visium, GeoMx DSP, MERFISH)	Regional/multicellular spatial resolution (10–100 µm spots)	Fresh-frozen or FFPE tissue; maintains tissue architecture	- Retains histological context - Enables region-specific gene expression mapping - Integrates morphology and molecular profiles	- Limited spatial resolution (spots may contain multiple cells) - High cost and low throughput - Requires advanced imaging and computational analysis
Liquid Biopsy Transcriptomics (Urine/Blood RNA-seq)	Bulk-level, dynamic, longitudinal sampling	Urine supernatant/sediment or peripheral blood; minimally invasive	- Enables non-invasive, repeatable monitoring - Captures dynamic transcriptomic changes over time - Facilitates clinical translation and biomarker development	- Potential confounding from systemic signals - Variable RNA yield and stability - Requires cross-validation with tissue-level data

## Data Availability

No new data were created or analyzed in this study. Data sharing is not applicable to this article.

## Abbreviations

The following abbreviations are used in this manuscript:

AKI	Acute kidney injury
AUC	Area under the receiver operating characteristic curve
C1q	Complement component 1q
C1QB	Complement C1q B chain
C3	Complement component 3
C4d	Complement component 4d
C5b-9	Complement component C5b-9
CCL2	C-C motif chemokine ligand 2
CD	Cluster of differentiation
CFB	Complement factor B
CFD	Complement factor D
CKD	Chronic kidney disease
COL1A1	Collagen type I alpha 1 chain
CSF1	Colony-stimulating factor 1
CSF1R	Colony-stimulating factor 1 receptor
CXCL10	C-X-C motif chemokine ligand 10 (also known as IP-10)
CXCL12	C-X-C motif chemokine ligand 12
CXCR4	C-X-C chemokine receptor 4
DEGs	Differentially expressed gene(s)
DNMT1	DNA methyltransferase 1
ECM	Extracellular matrix
eGFR	Estimated glomerular filtration rate
EMT	Epithelial-to-mesenchymal transition
ETS1	ETS proto-oncogene 1 transcription factor
FDR	False discovery rate
FFPE	Formalin-fixed paraffin-embedded
FKBP5	FK506 binding protein 5
FN1	Fibronectin 1
GBP1	Guanylate binding protein 1
GO	Gene Ontology
GZMB	Granzyme B
H&E	Hematoxylin and eosin
HAVCR1	Hepatitis A virus cellular receptor 1
HCK	Hematopoietic cell kinase
ICAM1	Intercellular adhesion molecule 1
IDI	Integrated discrimination improvement
IFNG	Interferon gamma
IFTA	Interstitial fibrosis and tubular atrophy
IgAN	IgA nephropathy
IL	Interleukin
JAK/STAT	Janus kinase/signal transducer and activator of transcription
JCHAIN	Joining chain of multimeric IgA and IgM
KEGG	Kyoto Encyclopedia of Genes and Genomes
KIM-1	Kidney injury molecule-1
KLRC1	Killer cell lectin like receptor C1
KPMP	Kidney Precision Medicine Project
LCM	Laser capture microdissection
LCN2	Lipocalin 2
MCP-1	Monocyte chemoattractant protein-1
MEST	Mesangial, Endocapillary, Segmental sclerosis, Tubular atrophy/interstitial fibrosis classification
MRC1	Mannose receptor C-type 1
mRNA	Messenger RNA
NF-κB	Nuclear factor kappa-light-chain-enhancer of activated B cells
NGAL	Neutrophil gelatinase-associated lipocalin
NK	Natural killer cell
NPHS1	Nephrin
NRI	Net reclassification improvement
PBMC	Peripheral blood mononuclear cell
PDCD1	Programmed cell death protein 1
PRRX1	Paired related homeobox 1
RIN	RNA Integrity Number
RNA-seq	RNA sequencing
S100A8/A9	S100 calcium-binding protein A8/A9
scRNA-seq	Single-cell RNA sequencing
snRNA-sea	Single-nucleus RNA sequencing
SYK	Spleen tyrosine kinase
TCF21	Transcription factor 21 (Pod-1, epicardi
TGF-β	Transforming growth factor-beta
TNF	Tumor necrosis factor
TYROBP	TYRO protein tyrosine kinase-binding protein
WGCNA	Weighted gene co-expression network analysis
